# Deprioritized and disrupted: tuberculosis care in the shadow of COVID-19

**DOI:** 10.3389/fpubh.2025.1651902

**Published:** 2025-11-07

**Authors:** Sushant Sharma

**Affiliations:** School of Public Health Sciences, Faculty of Health, University of Waterloo, Waterloo, ON, Canada

**Keywords:** tuberculosis, COVID-19, health systems, global health equity, public health disruption, India, Canada, Nigeria

## Abstract

The COVID-19 pandemic significantly disrupted tuberculosis (TB) care worldwide, undermining years of progress in TB prevention and control. This Perspective offers a comparative analysis of how TB services were affected in a high-income, low-burden country (Canada) versus two low- and middle-income, high-burden countries (India and Nigeria). Drawing on secondary data and global surveillance reports, the article highlights key disruptions across the TB care cascade, including delays in diagnosis, reduced case detection, and the collapse of community-based treatment models like DOTS. In Canada, digital transitions partially mitigated the impact, though access was unequal. In contrast, India and Nigeria faced widespread diagnostic interruptions, compounded by preexisting infrastructure gaps and limited digital access. The comparison reveals how underlying health system strength and digital readiness shaped national responses and recovery trajectories. Crucially, the pandemic exposed policy inertia and the deprioritization of routine infectious disease care in crisis contexts. This article calls for a global rethink of public health preparedness that centers on equity, continuity of essential services, and support for high burden settings. By analyzing divergent country experiences, this Perspective contributes actionable insights for strengthening TB programs and public health systems during future pandemics.

## Introduction

In the wake of the unprecedented global COVID-19 pandemic, the intricacies of healthcare delivery and its far-reaching implications have come under intense scrutiny (1). Among the many challenges the pandemic posed, the disruption of tuberculosis (TB) care emerged as a critical concern (2).

We selected India and Canada to represent opposite ends of a global spectrum in terms of TB burden, healthcare infrastructure, and available pandemic-related data on TB care. This comparative approach enables an analysis that captures both resource-constrained and resource-rich contexts. These examples offer boundary cases that allow us to draw lessons applicable to other nations that fall between them, particularly countries with lower TB burdens but high pandemic-related service disruptions.

The pandemic’s sweeping impact on healthcare systems globally necessitates a reflection on how TB care, particularly in high-burden countries like India and those across Africa, as well as low-burden countries such as Canada, was affected (3, 4).

COVID-19 exposed and deepened existing healthcare inequities. This commentary highlights the consequences of the pandemic on TB care, drawing attention to service disruptions, access limitations, and diverted public health resources.

## Impact of COVID-19 on TB care in high-burden countries

The pandemic brought severe restrictions on mobility in many African countries, which hindered patients’ ability to access healthcare. Community transmission of COVID-19 intersected with already fragile health systems, as seen in Nigeria, where statutory lockdowns triggered widespread closures of healthcare facilities. Drugstores and laboratories were less affected, suggesting the potential benefit of involving private-sector partners more actively in TB care (5).

Limited access to HIV and TB medications, sterile equipment, and contraception was reported, highlighting how structural barriers exacerbated health risks during lockdowns (6). World Health Organization’s guidance promoting multi-month dispensing and remote consultation was an attempt to mitigate some of these effects (7), but implementation varied significantly.

In India, which accounts for roughly a quarter of global TB cases, lockdowns significantly reduced TB case detection (8). Disruptions to immunization campaigns, including the BCG vaccine, introduced further risks (9). Healthcare providers also faced a lack of PPE, impeding their ability to provide routine TB care.

Closure of outpatient departments and difficulties accessing DOTS centers during lockdowns were commonly reported (8). For patients on multidrug-resistant TB regimens, such as injectable Amikacin or Streptomycin, treatment delivery was severely compromised due to logistical constraints and healthcare access barriers.

## Impact of COVID-19 on TB care in Canada

Despite Canada’s relatively low TB burden, its health systems were not immune to disruption. A study by Geric et al. (3) showed a 30–66% decrease in TB infection therapy initiation rates across three centers in Montreal and Toronto. These declines in service provision risked setting back progress in TB prevention (10).

The pandemic forced health services to prioritize COVID-19 responses, often at the expense of TB care (11). Preventive TB services were sidelined, based on the perception that they were less urgent than treatment. This division reflects an outdated dichotomy between public health and clinical medicine.

Indigenous populations in Canada faced additional challenges. Inadequate communication around social distancing and quarantine, combined with limited access to specialist care in northern regions, further strained TB detection and contact tracing efforts (12, 16). These inequities raise serious concerns about how health crises magnify vulnerabilities in underserved populations.

## Shared themes across settings

Across high- and low-burden countries, similar patterns emerged. Lockdowns hindered physical access to TB care. Fragile health systems, particularly in LMICs, were further weakened. Even in high-income countries like Canada, the diversion of public health resources and services delayed TB diagnoses and disrupted preventive efforts (13).

The pandemic also underscored the dangers of deprioritizing TB care. As long as global vaccine inequities persist, COVID-19 variants will continue to strain systems and drain attention from other public health needs, including TB (14).

## Conclusion

The impact of COVID-19 on TB care has been far-reaching, exposing long-standing weaknesses in both high- and low-burden settings. Lockdowns and resource diversion disrupted essential services, while patients, particularly in marginalized communities were left more vulnerable than ever (3, 7, 8).

This crisis demands a recalibration of public health priorities. TB services must be protected and integrated into pandemic response planning. Marginalized populations, including those in rural India and Indigenous communities in Canada, must be at the center of recovery strategies. The pandemic has made it clear: siloed approaches to health care are no longer viable in a world of intersecting crises (15).

While the systems in Canada and India differ in structure, such as universal publicly funded care in Canada versus a mix of public and private provision in India, the disruptions observed in both highlight shared systemic fragilities. In India, the pandemic strained an already overburdened public sector (9, 13). In Canada, delays emerged despite a strong health infrastructure, due to workforce shortages and the reprioritization of services (3, 12). These differences underscore that even robust systems can falter without strategic pandemic planning that integrates existing disease programs like TB (14). The healthcare system context shaped the form of disruption but not its inevitability. This highlights the need for resilient, integrated, and equity-oriented systems worldwide.

## Data Availability

The original contributions presented in the study are included in the article/supplementary material, further inquiries can be directed to the corresponding author.
